# Effect of neoadjuvant chemotherapy on platinum resistance in stage IIIC and IV epithelial ovarian cancer

**DOI:** 10.1097/MD.0000000000004797

**Published:** 2016-09-09

**Authors:** Yanlin Luo, Maria Lee, Hee Seung Kim, Hyun Hoon Chung, Yong Sang Song

**Affiliations:** a Department of Gynecologic Oncology, Affiliated Cancer Hospital of Zhengzhou University (Henan Cancer Hospital), Zhengzhou, China; b Department of Obstetrics and Gynecology, Seoul National University College of Medicine, Seoul, Korea; c Cancer Research Institute, Seoul National University College of Medicine, Seoul, Korea; d Major in Biomodulation, World Class University, Seoul National University, Seoul, Korea.

**Keywords:** epithelial ovarian cancer, interval debulking surgery, neoadjuvant chemotherapy, overall survival, platinum resistance

## Abstract

It remains controversial whether neoadjuvant chemotherapy (NACT) followed by interval debulking surgery (IDS) induces chemoresistance in advanced epithelial ovarian cancer (EOC) compared with primary debulking surgery (PDS). The aim of this study was to compare platinum-resistant recurrence following treatment with NACT-IDS or PDS in patients with stage IIIC and IV EOC.

We retrospectively reviewed the records of 341 patients who underwent PDS or NACT-IDS for Federation of Gynecology and Obstetrics stage IIIC or IV EOC between March 1990 and December 2010. Risk factors of platinum resistance, including NACT, postoperative residual tumor size, and various clinicopathological factors, were evaluated by univariate and multivariate logistic regression analyses. Survival analysis was performed by the Kaplan–Meier method and Cox regression modeling to measure overall survival (OS).

Of 341 patients, 58 (17.0%) underwent NACT-IDS and 283 (83.0%) were treated with PDS. Twenty-nine (50.0%) patients developed platinum-resistant disease at first relapse after NACT-IDS and 99 (35.0%) patients recurred after PDS (*P* = 0.033). In the multivariate logistic regression analyses, NACT-IDS and postoperative residual tumor mass >1 cm were risk factors for platinum-resistant recurrence (adjusted odds ratios 2.950 and 2.915; 95% confidence intervals [CIs] 1.572–5.537 and 1.780–4.771, *P* = 0.001 and 0.000, respectively). Postoperative residual tumor mass >1 cm and platinum-resistant disease were significantly correlated with shorter OS (adjusted hazard ratios 1.579 and 4.078; 95% CI 1.193–2.089 and 3.074–5.412, *P* = 0.001 and 0.000, respectively), whereas NACT-IDS did not extend OS.

NACT-IDS increases the risk of platinum-resistant recurrence in patients with stage IIIC and IV EOC.

## 1 Introduction

Epithelial ovarian cancer (EOC) is the most lethal malignancy of the female genital tract. A total of 21,290 new cases and 14,180 deaths from ovarian cancer are expected to occur in the United States in 2015.^[1]^ The high mortality is chiefly due to the fact that approximately 75% of patients are diagnosed at an advanced stage with widespread peritoneal lesions and the frequent acquisition of chemoresistance.^[2]^ Primary debulking surgery (PDS) followed by platinum-based chemotherapy is the standard treatment for advanced EOC. Neoadjuvant chemotherapy with interval debulking surgery (NACT-IDS) is an alternative treatment option for stage III and IV patients with unresectable, extensive tumors or poor performance status.^[3]^ The size of the postoperative residual tumor is one of the most important prognostic factors regardless of the timing of surgery.^[4]^

However, the benefit of NACT-IDS still remains debatable. Two randomized trials^[5,6]^ and a meta-analysis^[7]^ reported a significantly higher optimal debulking rate in the NACT-IDS group than the PDS group, but this did not confer any survival benefit. Others have shown that NACT induces platinum resistance in vitro.^[8]^ Consequently, we reasoned that the potential benefit of the increased optimal debulking rate from NACT may be mitigated by the induction of chemoresistance before IDS. To date, the possibility of NACT-induced chemoresistance is still unclear and published data are limited.^[9]^ Therefore, in the present study we compared the rate of platinum-resistant recurrence between the NACT-IDS and PDS groups for stage IIIC and IV EOC patients.

## 2 Patients and methods

### 2.1 Patients

This retrospective study was approved by the Institutional Review Board of the Seoul National University Hospital. All enrolled patients underwent primary or IDS mostly with adjuvant platinum-based chemotherapy. All surgical procedures were performed by gynecologic oncologists with the aim of optimal cytoreduction. Optimal debulking surgery was defined as the biggest diameter of residual disease ≤1 cm. NACT-IDS was administered to patients with bulky metastatic tumors or poor performance status. Patients who experienced exploratory laparotomy for diagnostic biopsy or oophorectomy without debulking were included in the IDS group. Diagnosis of recurrence was determined by the date of the first imaging study which showed any finding of recurrence. Overall survival (OS) was evaluated from diagnosis to the date of death of any cause, or to the date of last follow-up. “Platinum-resistant disease” was defined as disease that responded to primary platinum therapy and then progressed within 6 months of the last dose of primary platinum therapy, “platinum-sensitive disease” was defined as disease that relapsed 6 months or more after initial treatment, and “platinum-refractory disease” was defined as disease that progressed or was stable during platinum therapy.^[10]^ Clinical information was collected, including age, the Federation of Gynecology and Obstetrics stage, histological grade, subtype, postoperative residual tumor size, regimen and cycles of adjuvant chemotherapy, CA125 level at diagnosis, platinum-resistant disease, and OS.

### 2.2 Statistical analysis

Continuous variables were evaluated by the Student *t* test or an ANOVA test. Categorical variables were evaluated by the χ^2^ test or Fisher exact test. We performed logistic regression analyses with odds ratios (ORs) and 95% confidence intervals (CIs) for evaluating factors to reduce the risk of platinum resistance. Kaplan–Meier analyses were used to construct survival curves. Multivariate Cox models with hazard ratios (HRs) and 95% CIs were used for investigating factors for improved OS. For these analyses, we used SPSS software version 21.0 (SPSS Inc, Chicago, IL) and a *P* < 0.05 was considered to be statistically significant.

## 3 Results

### 3.1 Patient enrollment

All patient information for the current study was retrieved from a database of 370 patients who were diagnosed with histologically proven stage IIIC and IV EOC at Seoul National University Hospital between March 1990 and December 2010. Of these, we enrolled 341 patients who underwent primary or IDS with adjuvant chemotherapy. A total of 29 patients were excluded, including 4 patients that lacked data on the recurrence status, 15 patients with incomplete operation or chemotherapy data, and 10 patients who were lost to follow-up or the follow-up duration was <6 months.

### 3.2 Characteristics of the patients in the NACT-IDS and PDS groups

Of the 341 enrolled patients, 58 (17.0%) underwent NACT-IDS and 283 (83.0%) were treated with PDS. Table 1 shows the clinicopathological characteristics of the patients. Compared with the PDS group, patients in the NACT-IDS group had higher rate of stage IV diseases (41.4% vs 12.0%, *P* = 0.000) and postoperative residual tumor mass ≤1 cm (84.5% vs 46.3%, *P* = 0.000). The use of platinum- and paclitaxel-based chemotherapy was higher in the NACT-IDS group (96.6% vs 84.8%, *P* = 0.016), whereas the number of chemotherapy cycles was not significantly different between the 2 groups. Finally, the NACT-IDS and PDS groups had a similar distribution of patient age, histological grade, type, and CA125 level at diagnosis.

**Table 1:**
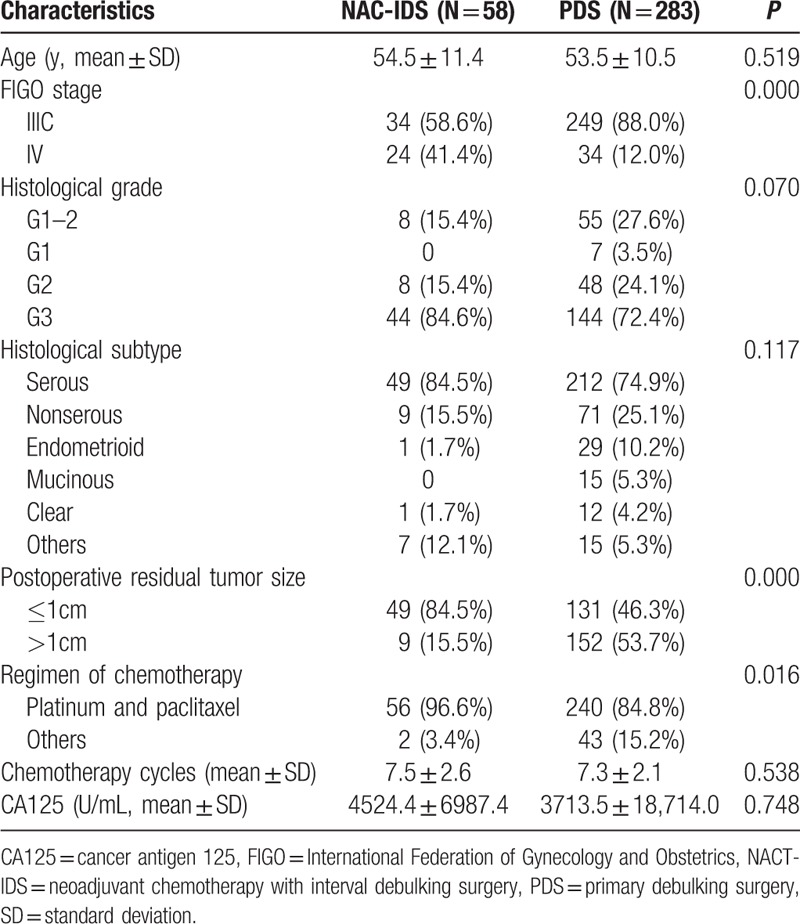
Comparision of clinicopathological characteristics between NACT-IDS and PDS group in 341 patients with stage IIIC and IV epithelial ovarian cancer.

### 3.3 Platinum-resistant recurrence analysis

During the follow-up period, 128 (37.5%) patients experienced platinum-resistant recurrence, 50.0% (29/58) of which were in the NACT-IDS group and 35.0% (99/283) of which were in the PDS group. As shown in Table 2, univariate logistic regression analyses found that postoperative residual tumor mass >1 cm and NACT-IDS were independent factors to increase the risk of platinum-resistant disease at first recurrence (ORs 2.201 and 1.859; 95% CI 1.408–3.441 and 1.051–3.288, *P* = 0.001 and 0.033, respectively). The variables with a *P* < 0.05 in the univariate analyses were included in the final multivariate logistic regression analyses. Postoperative residual tumor mass >1 cm and NACT-IDS were risk factors for increased platinum resistance (adjusted ORs 2.915 and 2.950; 95% CI 1.780–4.771 and 1.572–5.537, *P* = 0.000 and 0.001, respectively).

**Table 2:**
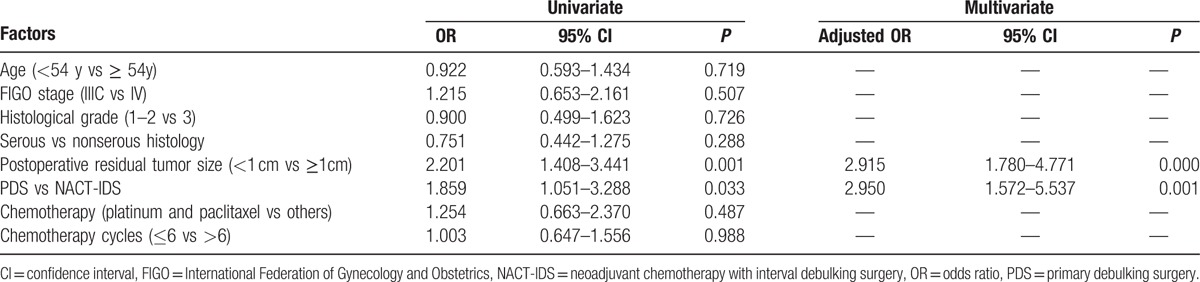
Univariate and multivariate analysis of risk factors for platinum resistance recurrence after NACT-IDS and PDS.

### 3.4 Survival analysis

**Figure 1:**
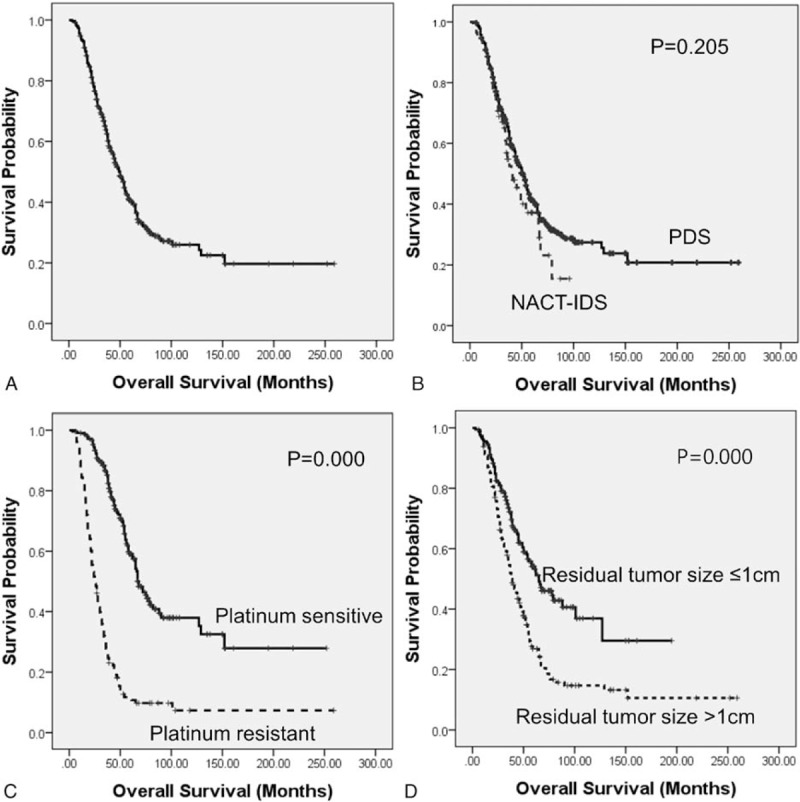
Kaplan–Meier survival analyses for overall survival between the NACT-IDS and PDS groups: (A) all patients; (B) NACT-IDS versus PDS; (C) platinum-resistant versus platinum-sensitive; (D) postoperative residual tumor size >1 cm versus ≤1 cm. All *P* values were calculated using the log-rank test. NACT-IDS = neoadjuvant chemotherapy with interval debulking surgery, PDS = primary debulking surgery.

Kaplan–Meier analysis and the log-rank test (Fig. 1) showed that the median OS was significantly worse in patients with postoperative residual tumor mass >1 cm and platinum resistance at the first recurrence. However, OS was not significantly different between the NACT-IDS and PDS groups. The total median OS was 50.0 months (95% CI 44.5–55.5 months) (Fig. 1A). The median OS was 41.0 months in the NACT-IDS group versus 51.0 months in the PDS group (*P* = 0.205; Fig. 1B), 25.0 months in the platinum-resistant group versus 68.0 months in the platinum-sensitive group (*P* = 0.000; Fig. 1C), and 39.0 months in the postoperative residual tumor mass >1 cm group versus 65.0 months in the postoperative residual tumor mass ≤1 cm group (*P* = 0.000; Fig. 1D).

In univariate survival analysis, postoperative residual tumor mass >1 cm, platinum resistance, and older age were significantly associated with unfavorable OS (HRs 1.947, 4.392, and 1.351; 95% CI 1.478–2.563, 3.326–5.800, and 1.030–1.771; *P* = 0.000, 0.000, and 0.030, respectively). However, tumor stage, histological grade, type, chemotherapy regimen, and number of cycles were not significantly related to OS in univariate survival analysis (Table 3). In multivariate survival analysis, postoperative residual tumor mass >1 cm and platinum resistance were independent risk factors to decrease OS (adjusted HRs 1.579 and 4.078; 95% CI 1.193–2.089 and 3.074–5.412, *P* = 0.001 and 0.000, respectively) (Table 3).

**Table 3:**
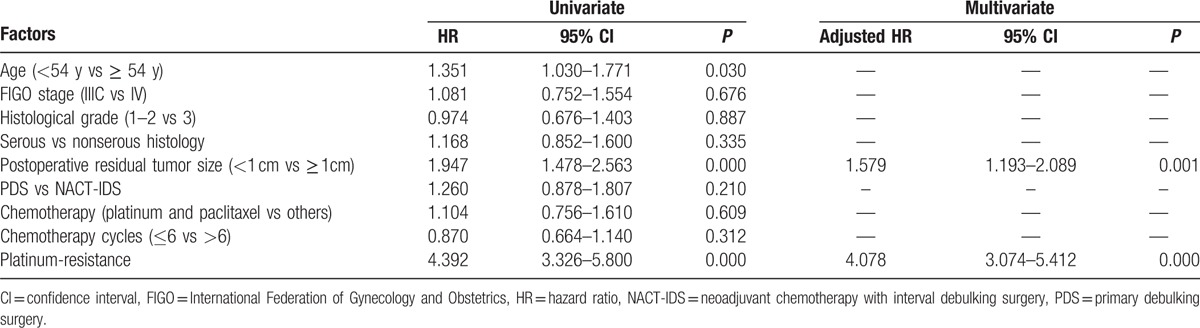
Univariate and multivariate Cox's proportional hazard analyses for evaluating overall survival in 341 patients with stage IIIC and IV epithelial ovarian cancer after NACT-IDS and PDS.

## 4 Discussion

For the past decade, there has been much controversy regarding the use of NACT for advanced EOC.^[11]^ Furthermore, it remains unclear whether NACT can induce platinum-resistant disease. Our study showed that the NACT-IDS group had a higher incidence of platinum-resistant disease at first relapse than the PDS group, which was also confirmed in the multivariate regression analysis (adjusted OR 2.950, *P* = 0.001). Three retrospective trials^[12–15]^ also demonstrated that patients in the NACT-IDS group had a higher risk of platinum-resistant recurrence than those who underwent PDS. However, in studies by Rauh-Hain^[12]^ and da Costa,^[13]^ no significant difference in platinum-resistant disease at the first relapse was observed, whereas the risk of platinum-resistant disease at the second relapse was higher in both Rauh-Hain's (adjusted OR 4.06, *P* = 0.001) and da Costa's (adjusted HR 1.92, *P* = 0.009) studies. Moreover, in Petrillo's and Rauh-Hain's studies,^[14]^ there was a significant difference in platinum-resistant disease at first recurrence only in the univariate analysis. Therefore, to the best of our knowledge, our study is the first to show that NACT-IDS is an independent risk factor for platinum-resistant disease at first relapse in stage IIIC and IV EOC.

A possible hypothesis to explain our result is that the larger the volume of cancer present when chemotherapy is initiated, the higher the likelihood of development of mutations and chemoresistance.^[15,16]^ According to this theory, patients receiving NACT are susceptible to developing mutant cells and acquiring chemoresistance due to the exposure of a larger tumor burden to chemotherapy before IDS. Other studies have been done that support our result. For instance, Lim et al^[17]^ suggested that NACT enriches for cancer stem cells, which leads to chemoresistance. Another study reported that the *TP53* K351N mutation may induce platinum resistance after platinum-based NACT and act as an independent risk factor for shorter disease-free survival in advanced ovarian cancer patients who underwent NACT-IDS.^[18]^ Postoperative residual tumor mass >1 cm is another risk factor associated with higher platinum-resistant recurrence risk (adjusted OR 2.915, *P* = 0.000), which is consistent with Rauh-Hain's research.^[19]^

In survival analyses, we found that 2 variables, postoperative residual tumor mass >1 cm and platinum-resistant disease, were significantly correlated with shorter OS. It is well established that optimal debulking surgery is one of the most important independent prognostic factors,^[20]^ and platinum-resistant disease has a lower response rate to secondary chemotherapy and has a poor prognosis.^[21]^ However, the NACT-IDS group had no extension of OS versus PDS group (median OS: 41.0 months vs 51 months, *P* = 0.205), which is similar to previous results.^[5–7]^ We reasoned that the potential extension of OS from the higher rate of optimal debulking surgery in the NACT-IDS group was partially mitigated by the platinum resistance induced by NACT. Moreover, the higher rate of stage IV patients enrolled in the NACT-IDS group is consistent with the indications for the use of NACT-IDS.^[19]^

The current study is relatively larger than similar studies performed on this topic. Our research provides more significant data to demonstrate the relationship between platinum-resistant disease and NACT-IDS. However, there were some limitations to our study. For instance, the retrospective chart review may have unmeasured confounders. Selection bias for NACT-IDS is a very important factor for data analysis and it contains a relatively small number of patients in NACT-IDS group; prospective study is needed to derive the correct conclusion.

## 5 Conclusions

In conclusion, NACT-IDS may increase the risk of platinum resistance in stage IIIC and IV EOC. Further studies are needed to explore the possible mechanism of acquired platinum resistance so that it may be circumvented or reversed. Furthermore, during the development of a treatment strategy, platinum resistance should be taken into consideration before the use of NACT-IDS in advanced EOC patients.
